# Upregulation of Myelin Gene Expression by a Physically-Modified Saline via Phosphatidylinositol 3-Kinase-Mediated Activation of CREB: Implications for Multiple Sclerosis

**DOI:** 10.1007/s11064-017-2435-1

**Published:** 2017-11-15

**Authors:** Malabendu Jana, Supurna Ghosh, Kalipada Pahan

**Affiliations:** 1 Department of Neurological Sciences, Rush University Medical Center, 1735 West Harrison St, Suite 310, Chicago, IL 60612 USA; 2 Revalesio Corporation, 1200 East D Street, Tacoma, WA 98421 USA

**Keywords:** Physically-modified saline, Oligodendrocytes, Myelin genes, PI-3 kinase, CREB

## Abstract

An increase in central nervous system (CNS) remyelination and a decrease in CNS inflammation are important steps to halt the progression of multiple sclerosis (MS). RNS60 is a bioactive aqueous solution generated by subjecting normal saline to Taylor–Couette–Poiseuille flow under elevated oxygen pressure. Recently we have demonstrated that RNS60 exhibits anti-inflammatory properties. Here, we describe promyelinating property of RNS60. RNS60, but not normal saline (NS), RNS10.3 (TCP-modified saline without excess oxygen) or PNS60 (saline containing excess oxygen without TCP modification), stimulated the expression of myelin-specific genes and proteins (myelin basic protein, MBP; myelin oligodendrocyte glycoprotein, MOG and proteolipid protein, PLP) in primary mouse oligodendroglia and mixed glial cells. While investigating the mechanisms, we found that RNS60 treatment induced the activation of cAMP response element binding protein (CREB) in oligodendrocytes, ultimately leading to the recruitment of CREB to the promoters of myelin-specific genes. Furthermore, activation of type 1A p110β/α, but not type 1B p110γ, phosphatidylinositol-3 (PI-3) kinase by RNS60 together with abrogation of RNS60-mediated activation of CREB and upregulation of myelin genes by LY294002 (a specific inhibitor of PI-3 kinase) suggest that RNS60 upregulates the activation of CREB and the expression of myelin-specific molecules in oligodendrocytes via activation of PI3 kinase. These results highlight a novel promyelinating property of RNS60, which may be of benefit for MS and other demyelinating disorders.

## Introduction

Myelination is a complex process that requires proliferation of oligodendrocytes progenitors, migration to various brain regions, differentiation, and finally, synthesis and deposition of the myelin membrane. A number of neurologic disorders (multiple sclerosis, spinal cord injury, X-linked adrenoleukodystrophy, adrenomyeloneuropathy, neurotrauma, and HIV encephalomyelopathy) are associated with loss and/or dysfunction of the myelin sheath. The disabling burden of these neurodegenerative diseases has triggered a growing interest in gaining a better understanding of the molecular mechanisms regulating myelination [1–6].

Oligodendrocytes are the only specialized myelin-synthesizing cells in the CNS that express several myelin-specific genes like myelin basic protein (MBP), 2′,3′-cyclic nucleotide 3′-phosphodiesterase (CNPase), myelin oligodendrocyte glycoprotein (MOG), and proteolipid protein (PLP) [4, 5, 7–10]. In addition, oligodendrocytes also express galactosylceramidase (GalC) that is essential for the unperturbed lipid bilayer of the myelin membrane [11]. Moreover, it has been reported that an increase in MBP, MOG and PLP expression is a prerequisite to maturation of oligodendrocytes [12]. Therefore, increases in the expression of myelin-specific genes are essential for CNS remyelination. However, despite intense investigation, very few drugs are available that may increase or support the expression of myelin genes and help in remyelination.

Recently we have demonstrated that saline (0.9% sodium chloride or NaCl) processed by Taylor–Couette–Poiseuille (TCP) turbulence in the presence of elevated oxygen pressures attains anti-inflammatory property [13]. It increases the level of IκBα, inhibits the activation of NF-κB and suppresses the expression of pro-inflammatory molecules in microglia [13]. Our studies have also shown that RNS60 is capable of suppressing the disease process in animal models of multiple sclerosis [14, 15], Parkinson’s disease [16] and Alzheimer’s disease [17]. Others [18] have also reported upregulation of synaptic transmission by RNS60 via increased adenosine triphosphate (ATP) synthesis. Here, we describe that RNS60 is capable of increasing the expression of myelin-specific genes in primary mouse oligodendrocytes and mixed glial cells. RNS60 induces the activation of type IA phosphatidylinositol-3 kinase (PI3K) and increases the transcription of myelin genes via PI3K-mediated activation of cAMP response element binding protein (CREB), suggesting the possible use of RNS60 in remyelination.

## Materials and Methods

### Reagents

Fetal bovine serum, Hank’s balanced salt solution (HBSS), trypsin, and DMEM/F-12 were from Mediatech (USA). Antibiotic–antimycotic mixture and poly-d-lysine were obtained from Sigma-Aldrich (St. Louis, MO).

### Preparation of RNS60

RNS60 was generated at Revalesio (Tacoma, WA) using Taylor–Couette–Poiseuille (TCP) flow as described before [13, 16]. Briefly, sodium chloride (0.9%) for irrigation, USP pH 5.6 (4.5–7.0, Hospira), was processed at 4 °C and a flow rate of 32 mL/s under 1 atm of oxygen back-pressure (7.8 mL/s) gas flow rate, while maintaining a rotor speed of 3450 rpm. Chemically, RNS60 contains water, sodium chloride, 50–60 parts/million oxygen, but no active pharmaceutical ingredients.

Following controls for RNS60 were also used in this study: (a) NS, normal saline from the same manufacturing batch. This saline contacted the same device surfaces as RNS60 and was bottled in the same way, (b) RNS10.3, saline that was processed through the same device without adding excess oxygen, and (c) PNS60, saline with same oxygen content (55 ± 5 ppm) that was prepared inside of the same device but was not processed with TCP flow. Careful analysis demonstrated that these fluids were chemically identical [13, 14]. Liquid chromatography quadrupole time-of-flight mass spectrometric analysis also showed no difference between RNS60 and other control solutions [13]. On the other hand, by using atomic force microscopy (AFM), we studied nanobubble nucleation in RNS60 and other saline solutions and observed that RNS60 has a nanobubble composition different from that of control saline solutions [13]. This same relative pattern of nanobubble number and size was observed when positive potentials were applied to AFM surfaces with the same control solutions, suggesting the involvement of charge in stabilization of nanobubbles in RNS60 [13].

### Isolation of Mouse Mixed Glial Cultures and Primary Oligodendrocytes

Oligodendrocytes were isolated as described before [3–5, 7]. Briefly, brains isolated from 2 to 3 day-old mouse pups were dissociated by trituration and trypsinization (0.25% trypsin in PBS at 37 °C for 15 min). The trypsin was inactivated with 10% heat-inactivated FBS (Mediatech, Washington, DC). The dissociated cells were filtered successively through 380 and 140 µm meshes (Sigma, St. Louis, MO) and pelleted by centrifugation. The resulting suspension was centrifuged for 10 min at 1500 rpm and then re-suspended in DMEM/F12 supplemented with 20% heat inactivated FBS. Cells were plated on poly-d-lysine-coated 75 cm^2^ flasks and incubated at 37 °C with 5% CO_2_ in air. Culture medium was changed after every 3 days. The initial mixed glial cultures, grown for 9 days, were placed on a rotary shaker at 240 rpm at 37 °C for 2 h to remove loosely attached microglia. The oligodendrocytes were detached after shaking for 18 h at 200 rpm at 11 days. To purify oligodendrocytes from astrocytes and microglia, the detached cell suspension was plated in tissue culture dishes (2 × 10^6^ cells/100 mm) for 60 min at 37 °C. This step was repeated twice for non-adherent cells to minimize the contamination. The non-adhering cells, mostly oligodendrocytes, were seeded onto poly-d-lysine-coated culture plates in complete medium (DMEM/F12 supplemented with 10% heat inactivated FBS) at 37 °C with 5% CO_2_ in air. The remaining cells in the flask are astrocytes. Previously we [4, 5] have shown that oligodendrocytes isolated through this procedure are more than 98% pure.

### Treatment of Primary Oligodendrocytes with RNS60

Cells were initially cultured on poly-d-lysine-coated plates in DMEM/F-12 containing 10% FBS for 24 h. Then cells were treated with different concentrations of RNS60, NS, PNS60, and RNS10.3 under serum-free conditions.

### Immunostaining

Immunostaining was performed as described earlier [4, 5, 19]. Briefly, coverslips containing 200–300 cells/mm^2^ were fixed with 4% paraformaldehyde for 15 min, followed by treatment with cold ethanol (− 20 °C) for 5 min and two rinses in PBS. Samples were blocked with 3% BSA in PBS containing Tween 20 (PBST) for 30 min and incubated in PBST containing 1% BSA and rabbit anti-MOG (1:100), rabbit anti-GalC (1:200) or goat anti-PLP (1:200) (Table 1). After three washes in PBST (15 min each), slides were further incubated with Cy5 and Cy2 (Jackson ImmunoResearch, West Grove, PA). For negative controls, a set of culture slides was incubated under similar conditions without the primary antibodies. The samples were mounted and observed under a Bio-Rad MRC1024ES confocal laser-scanning microscope.

**Table 1:** Antibodies used in this study

Antibody	Manufacturer	Catalog	Host	Application	Dilution
PLP	Santa Cruz	Sc-23570	Goat	WB, IF	1:1000 (WB) 1:200 (IF)
MOG	Abcam	Ab109746	Rabbit	WB, ChIP, IF	1:1000 (WB) 1:100 (IF) 2 µg (ChIP)
MBP	Santa Cruz	SC-13914	Goat	WB, ChIP	1:1000 (WB) 2 µg (ChIP)
GalC	Sigma	G9152	Rabbit	IF	1:200
GFAP	Santa Cruz	SC-6171	Goat	WB	1:1000
p-CREB	Cell Signaling	9198L	Rabbit	WB	1:500
CREB	Cell Signaling	9197S	Rabbit	WB	1:500
p110 α	Santa Cruz	SC-293172	Mouse	WB	1:1000
p110 β	Santa Cruz	SC-602	Rabbit	WB	1:1000
p110 γ	Santa Cruz	SC-7177	Rabbit	WB	1:1000
β-Actin	Abcam	Ab6276	Mouse	WB	1:6000
CBP	Santa Cruz	SC-369	Rabbit	ChIP	2 µg
p300	Santa Cruz	SC-585	Rabbit	ChIP	2 µg
CREB	Millipore	CS203204	Rabbit	ChIP	2 µg
IgG	Santa Cruz	SC-3888	Rabbit	ChIP	2 µg

*IF* immunofluorescence, *WB* western blot, *ChIP* Chromatin immunoprecipitation

### Semi-quantitative Polymerase Chain Reaction (RT-PCR) Analysis

Total RNA was isolated from mouse oligodendrocytes and mixed glial cells using RNA-Easy Qiagen kit following manufactures protocol. To remove any contaminating genomic DNA, total RNA was digested with DNase. Semi-quantitative RT-PCR was carried out as described previously using oligo(dT)_12−18_ as primer and MMLV reverse transcriptase (Clontech) in a 20-μl reaction mixture [5, 19]. The resulting cDNA was appropriately diluted, and diluted cDNA was amplified using Titanium Taq polymerase and the following primers.

Mouse (m) MOG: sense, 5′-CCT CTC CCT TCT CCT CCT TC-3^′^; antisense, 5′-AGA GTC AGC ACA CCG GGG TT-3′; mPLP: sense, 5′-CTT CCC TGG TGG CCA CTG GAT TGT-3′; antisense, 5′-CCG CAG ATG GTG GTC TTG TAG TCG-3′; mMBP; Sense: 5′-TGG AGA GAT TCA CCG AGG AGA GGC-3′; antisense: 5′-TGA AGC TCG TCG GAC TCT GAG GGC-3′; mGAPDH: sense, 5′-CAG GGG ATG ATC ATG GCT TCT CC-3′; antisense, 5′-GAT GCT CAC AAG AGC CCC GTT AGC-3′. Amplified products were electrophoresed on a 1.8% agarose gel and visualized by ethidium bromide staining. Message for the GAPDH (glyceraldehyde-3-phosphate dehydrogenase) gene was used to ascertain that an equivalent amount of cDNA was synthesized from different samples.

### Real-Time PCR Analysis

It was performed using the ABI-Prism7700 sequence detection system (Applied Biosystems, Foster City, CA) as described earlier [5, 19–21]. All primers and FAM-labeled probes for mouse MBP, PLP, MOG, and GAPDH were obtained from Applied Biosytems. The mRNA expression of myelin genes was normalized to the label of GAPDH mRNA. Data were processed by the ABI Sequence Detection System 1.6 software and analyzed by ANOVA.

### Immunoblotting

Western blotting was conducted as described earlier [7, 19, 21–23]. Briefly, cells were scraped in lysis buffer, transferred to microfuge tube and spun into pellets. The supernatant was collected, and was analyzed for protein concentration via the Bradford methods (Bio-Rad). SDS sample buffer was added to protein samples followed by boiling for 5 min. Denatured samples were electrophoresed on NeuPAGE Novex 4–12% Bis Tris gel (Invitrogen) and protein transferred onto a nitrocellulose membrane (Bio-Rad) using the Thermo-Pierce Fast Semi-Dry Blotter. The membrane was washed for 15 min in PBS plus Tween 20 (PBST) and blocked for 1 h in PBST containing 2% BSA. Next, membranes were incubated overnight at 4 °C under shaking conditions with primary antibodies against GalC, phospho-CREB, MOG, PLP, MBP, GFAP, p110α, p110β, p110γ, and β-actin (Table 1). The next day membranes were washed in PBST for 1 h, incubated in secondary antibodies for 1 h at room temperature, washed for one more hour and visualized under the Odyssey Infrared Imaging system (Li-Cor, Lincoln, Nebraska).

### Densitometric Analysis

Protein blots were analyzed using ImageJ (NIH, Bethesda, MD) and bands were normalized to their respective actin loading controls. Data are representative of the average fold change with respect to control for three independent experiments.

### Chromatin Immunoprecipitation (ChIP) Assay

Recruitment of CREB to MOG and MBP gene promoters was determined by ChIP assay as described earlier [23–25]. Briefly, oligodendrocytes were treated with RNS60 under serum free conditions and after 2 h of stimulation, cells were fixed by adding formaldehyde (1% final concentration), and cross-linked adducts were resuspended and sonicated. ChIP was performed on the cell lysate by overnight incubation at 4 °C with 2 µg of anti-CREB, anti-CBP, anti-RNA Polymerase II, or anti-p300 antibodies followed by incubation with protein G agarose (Santa Cruz Biotechnology) for 2 h. The beads were washed and incubated with elution buffer. To reverse the cross-linking and purify the DNA, precipitates were incubated in a 65 °C incubator overnight and digested with proteinase K. DNA samples were then purified, precipitated, and precipitates were washed with 75% ethanol, air-dried, and resuspended in TE buffer. Following primers were used for amplification of chromatin fragments of human MOG and MBP genes.

MOG promoter (spanning distal CREB; 237 bp):

- Sense: 5′-CTA GAT ACA TTT TTC TGT TTC AGC CTG-3′.
- Antisense: 5′-AGT CTG ACT GGG GTG AAA TAG AAT CTC-3′.

MOG promoter (spanning proximal CREB; 166 bp):

- Sense: 5′-TGC TCC AGT GCT GTG AGG GGT TGG GT-3′.
- Antisense: 5′-CTG GGA GGG CAC TAC TGC CCA AGC CC-3′.

Mouse MBP promoter (spanning distal CREB; 174 bp):

- Sense: 5′-GGG CAG GTG GAC AAG GTG GGG GTG-3′.
- Antisense: 5′-GAC AAC CCT TCC AGT CTA TCA CCC-3′.

Mouse MBP promoter (spanning proximal CREB; 228 bp):

- Sense: 5′-GGG CTC TCA GGC CAT CGC CCT CTG-3′.
- Antisense: 5′-GGG CCT GTA CGA GGC CTA GAG GGG-3′.

### Electrophoretic Mobility Shift Assay (EMSA)

Nuclear extracts were prepared, and EMSA was performed as described previously [7, 26, 27] with some modifications. Briefly, IRDye infrared dye end-labeled oligonucleotides containing the consensus binding sequence for CREB were purchased from Licor Biosciences. Six micrograms of nuclear extract was incubated with binding buffer and with infrared-labeled probe for 20 min. Subsequently, samples were separated on a 6% polyacrylamide gel in 0.25× TBE buffer (Tris borate-EDTA) and analyzed by the Odyssey Infrared Imaging System (LI-COR Biosciences).

### Statistical Analysis

All values are expressed as the mean ± SD of three independent experiments. Differences among means were analyzed using one-way ANOVA followed by Tukey’s post-hoc test. Wherever appropriate, statistical differences between means were also calculated by the Student’s *t* test. A *p* value of less than 0.05 (*p* < 0.05) was considered statistically significant.

## Results

### Upregulation of Myelin Gene Expression by RNS60 in Primary Mouse Oligodendrocytes

**Fig. 1:**
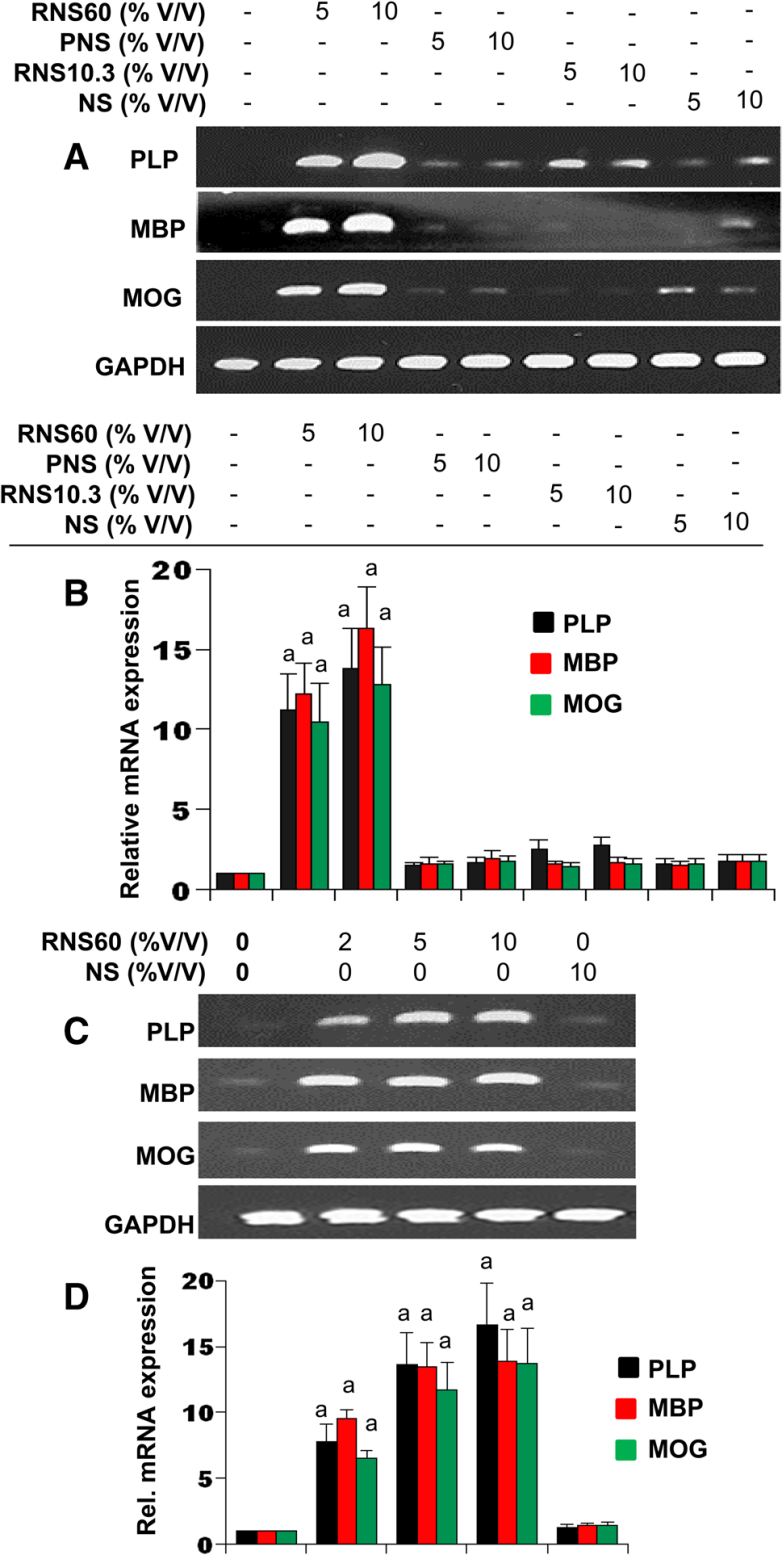
Effect of RNS60 on the expression of myelin genes in primary mouse oligodendrocytes. Cells were treated with RNS60, PNS, RNS10.3, and NS for 6 h under serum free condition followed by analysis of MBP, PLP and MOG mRNAs by semi-quantitative RT-PCR (**a**) and quantitative real-time PCR (**b**). Results are means ± SD of three different experiments. *p* < 0.005 vs. control. Cells were treated with different concentrations (2, 5, 10% v/v) of RNS60 for 6 h under serum free condition followed by analysis of MBP, PLP and MOG mRNAs by semi-quantitative RT-PCR (**c**) and quantitative real-time PCR (**d**). Results are mean ± SD of three different experiments. ^a^ *p* < 0.001 vs. control

One of the prerequisites of enhanced oligodendroglial differentiation is increased expression of myelin specific genes (e.g. MBP, MOG, CNPase, and PLP) in oligodendrocytes [6, 12, 28, 29]. Therefore, to investigate whether RNS60 containing charge-stabilized nano-structures induces the expression of myelin-specific genes in primary mouse oligodendrocytes, cells were treated with RNS60, PNS60, RNS10.3, or NS for 6 h. The expression of MBP, MOG and PLP was analyzed by semi-quantitative RT-PCR (Fig. 1a) and real-time PCR (Fig. 1b). Marked increase in the mRNA expression of MBP, MOG and PLP was observed by RNS60 treatment (Fig. 1a, b). However, unprocessed saline (NS) from the same manufacturing batch remained unable to increase the myelin gene expression in oligodendrocytes (Fig. 1a, b). Furthermore, under same treatment conditions, RNS10.3, saline that was processed with TCP flow in the absence of any excess oxygen, and PNS60, saline with same oxygen content as RNS60 (55 ± 5 ppm) that was exposed to the same device but not processed with TCP flow, also did not increase the mRNA expression of MBP, MOG and PLP in oligodendrocytes (Fig. 1a, b), indicating the specificity of the effect.

Semi-quantitative RT-PCR results in Fig. 1c clearly show that RNS60 at different doses increased the mRNA expression of MBP, MOG and PLP in primary oligodendrocytes. Again, NS had no effect on the mRNA expression of these myelin genes (Fig. 1c). Quantitative real time PCR data in Fig. 1d also support this conclusion. Significant (*p* < 0.001 vs. control) increase in the mRNA expression of MBP, MOG and PLP was observed even at the lowest dose (2% v/v) of RNS60 and the increase was maximum at a dose of 5 or 10% v/v RNS60 (Fig. 1c, d).

**Fig. 2:**
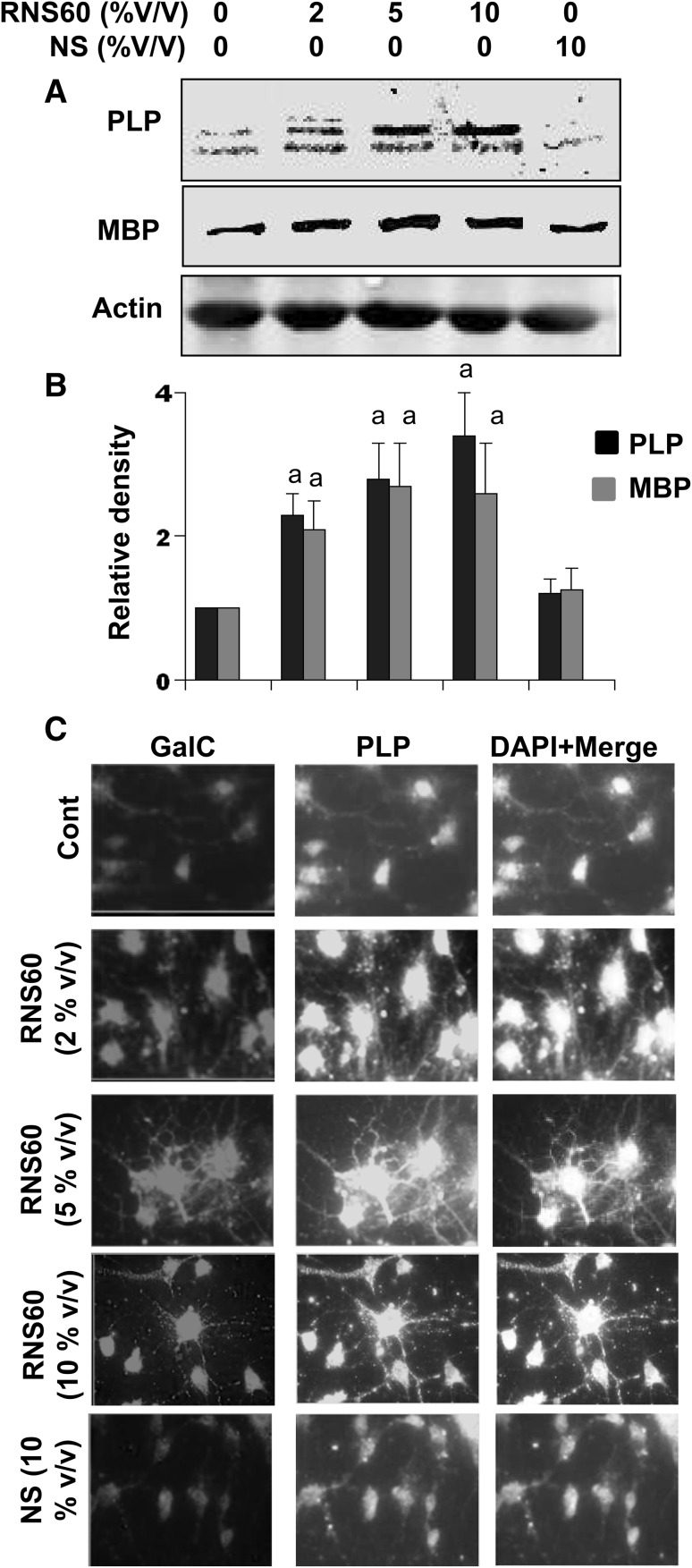
Effect of RNS60 on the expression of myelin-specific proteins in primary mouse oligodendrocytes. Cells were treated with different concentrations (2, 5, 10% v/v) of RNS60 for 18 h under serum free condition followed by monitoring the protein level of PLP and MBP by western blot (**a**). Actin was run as loading control. Bands were scanned and values (PLP/actin and MBP/actin) presented as relative to control (**b**). Results are mean ± SD of three different experiments. ^a^ *p* < 0.001 vs. control. Cells were also double-labeled with antibodies against GalC and PLP (**c**). The microscope setting was kept unaltered during the entire study. Figures are representative of three independent experiments

Next, we determined the effect of RNS60 on the expression of myelin proteins in primary oligodendrocytes. Similar to mRNA expression, RNS60, but not NS, increased the protein expression of MBP and PLP with significant (*p* < 0.001 vs. control) increase seen at a dose of 2% v/v RNS60 (Fig. 2a, b). Again, increases in myelin proteins by RNS60 were almost the same at 5 and 10% v/v RNS60 (Fig. 2a, b). Double-label immunofluorescence analysis also showed that RNS60, but not NS, markedly increased the levels of GalC and PLP at different doses in primary mouse oligodendrocytes (Fig. 2c). Taken together, these results suggest that RNS60 is capable of enhancing the expression of myelin-specific genes and proteins in primary mouse oligodendrocytes.

### Effect of RNS60 on the Expression of Myelin Genes in Mouse Mixed Glial Cultures

**Fig. 3:**
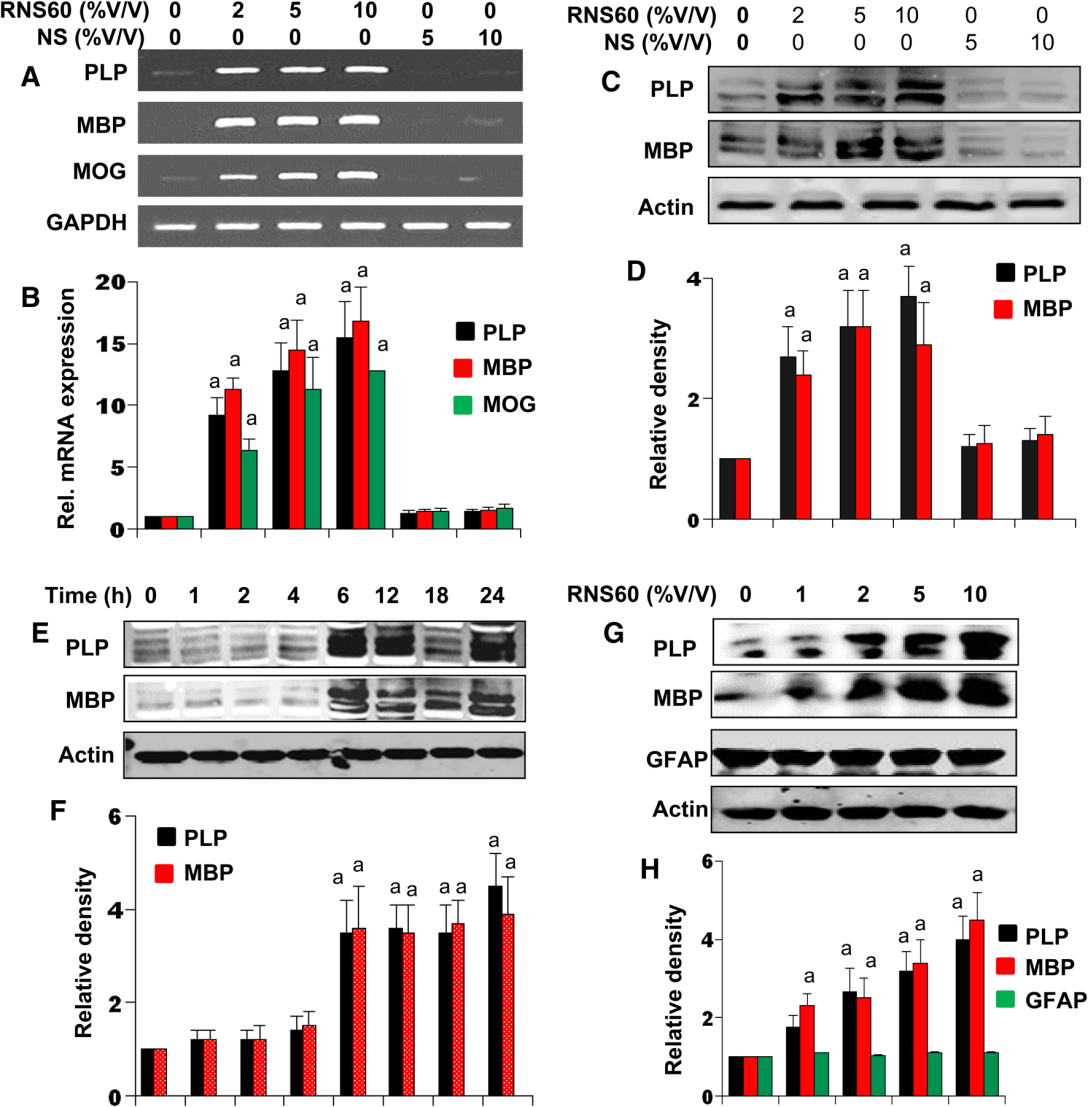
RNS60 induces the expression of myelin genes and proteins in mouse mixed glial cells in dose- and time-dependent manner. Cells were treated with different concentrations of RNS60 or NS for 6 h under serum free condition followed by analysis of MBP, PLP and MOG mRNAs by semi-quantitative RT-PCR (**a**) and quantitative real-time PCR (**b**). After 18 h of RNS60 or NS treatment, the protein level of PLP and MBP was examined by Western blot (**c**). Actin was run as loading control. Bands were scanned and values (PLP/Actin and MBP/actin) are presented as relative to control (**d**). Results are mean ± SD of three different experiments. ^a^ *p* < 0.001 vs. control. Cells were treated with 10% v/v of RNS60 for different time intervals followed by monitoring the protein level of MBP and PLP by western blot (**e**). Bands were scanned and values (PLP/Actin and MBP/actin) are presented as relative to control (**f**). After 18 h of RNS60 treatment, the protein level of GFAP, PLP and MBP was examined by western blot (**g**). Bands were scanned and values (PLP/Actin, MBP/actin and GFAP/Actin) are presented as relative to control (**h**). Results are means ± SD of three different experiments. ^a^ *p* < 0.001 vs. control

Next, we examined if RNS60 was capable of enhancing the expression of myelin genes in mouse mixed glial cultures. It is clearly evident from semi-quantitative RT-PCR (Fig. 3a) and real-time PCR (Fig. 3b) that RNS60, but not NS, was capable of enhancing the mRNA expression of MOG, PLP and MBP at different doses in mixed glial cells. Western blot analysis also showed that RNS60, but not NS, significantly (*p* < 0.001 vs. control) increased the protein expression of MBP and PLP in mixed glial cells at different doses (Fig. 3c, d). Time-dependent analysis showed that RNS60 was capable of increasing the protein expression of PLP and MBP significantly (*p* < 0.001 vs. control) as early as 6 h (Fig. 3e, f). However, we did not observe any further increase in subsequent hours of stimulation (Fig. 3e, f).

Astrocytes are the major cell type in the brain as well as in mixed glial cells. Therefore, to understand the specificity of the effect, we also monitored the effect of RNS60 on astrocytes. Since glial fibrillary acidic protein (GFAP) is the most important marker of astrocytes, we examined the effect of RNS60 on astrocytes. In contrast to the stimulation of oligodendroglia-specific proteins (PLP and MBP), RNS60 had no effect on the expression of GFAP protein in mixed glial cells (Fig. 3g, h). These results clearly suggest that RNS60 is capable of stimulating the expression of myelin-specific proteins in oligodendroglia in the presence of other brain cells and that the effect of RNS60 is specific for oligodendroglia.

### RNS60 Requires cAMP Response Element Binding Protein (CREB) for the Up-Regulation of Myelin Genes in Oligodendrocytes

**Fig. 4:**
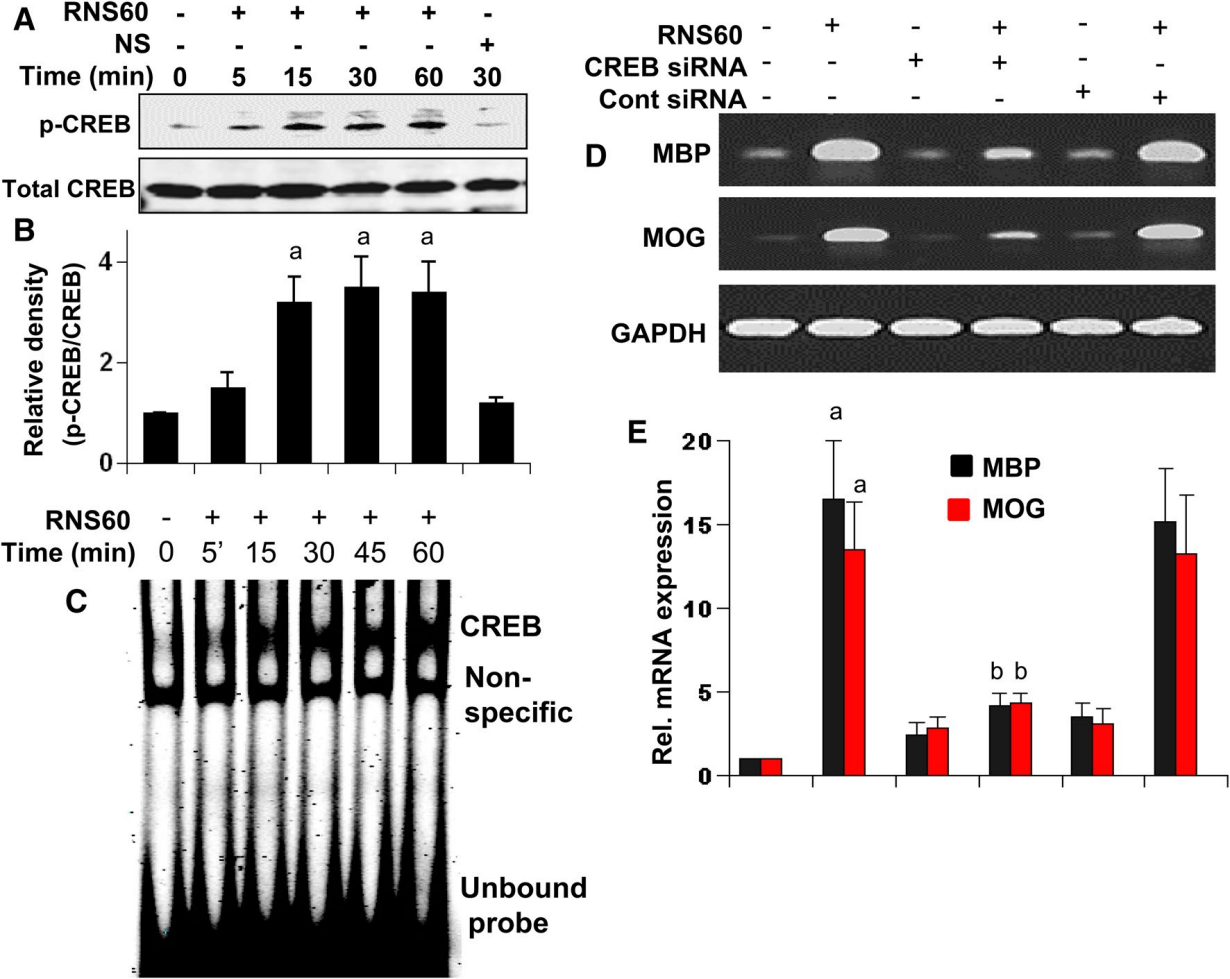
Role of CREB in RNS60-mediated increase in myelin genes in primary mouse oligodendrocytes. After different minutes of stimulation with 10% v/v RNS60 or NS under serum free conditions, cell lysates were prepared and analyzed by Western blotting with antibodies against phospho-CREB and total CREB (**a**). Bands were scanned and values (P-CREB/CREB) are presented as relative to control (**b**). Results are means ± SD of three different experiments. ^a^ *p* < 0.001 vs. control. After different minutes of stimulation with 10% v/v RNS60 under serum free conditions, nuclear extracts were examined for EMSA (**c**). Results represent three independent experiments. Cells were transfected with CREB siRNA and control siRNA using Lipofectamine Plus and nupherin reagent. After 24 h of transfection, cells were treated with 10% v/v RNS60 under serum-free condition for 6 h followed by monitoring the mRNA expression of MBP and MOG by semi-quantitative RT-PCR (**d**) and real-time-PCR (**e**). Results are mean ± SD of three separate experiments. ^a^ *p* < 0.001 vs. control; ^b^ *p* < 0.001 vs. control-RNS60

Next, we investigated mechanisms by which RNS60 increased the expression of myelin genes in oligodendrocytes. Since CREB plays an important role in the expression of different myelin-specific molecules [30, 31], we examined the role of CREB. At first, we monitored if RNS60 alone was capable of inducing the activation of CREB in primary oligodendrocytes. Activation of CREB was assessed by monitoring CREB phosphorylation and EMSA. RNS60, but not NS, induced the phosphorylation of CREB as depicted by Western blot of phospho-CREB and total CREB (Fig. 4a, b). RNS60 increased the level of phospho-CREB, but not total CREB, in oligodendrocytes significantly (*p* < 0.001 vs. control) at 15 min of stimulation (Fig. 4a, b). However, any further increase in the level of phospho-CREB was not seen at subsequent mins of RNS60 stimulation (Fig. 4a, b). To further bolster this result, we performed EMSA in order to monitor DNA-binding activity of CREB. As evident from Fig. 4c, RNS60 treatment led to time-dependent increase in DNA-binding activity of CREB with induction observed as early as 5 min. Next, to investigate whether CREB is involved in RNS60-mediated up-regulation of myelin gene expression, we employed CREB siRNA. Abrogation of RNS60-mediated up-regulation of MBP and MOG expression (Fig. 4d, e) by CREB siRNA, but not control siRNA, suggests that RNS60 increases the expression of myelin genes via CREB signaling pathway.

### RNS60 Induces the Recruitment of CREB to the Promoters of MBP and MOG Genes in Primary Mouse Oligodendrocytes

**Fig. 5:**
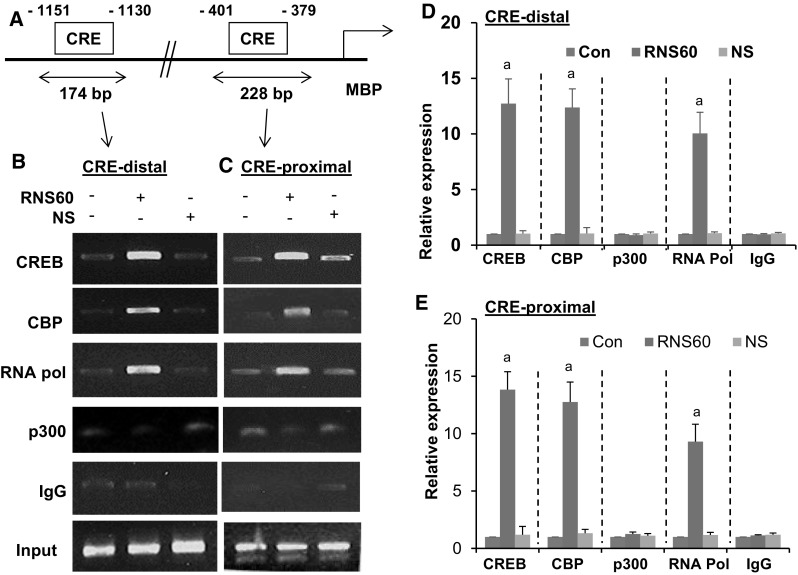
RNS60 treatment induces the recruitment of CREB to the MBP gene promoter in primary mouse oligodendrocytes. **a** Positions of two CREs in the mouse MBP gene promoter are shown. Cells were treated with 10% v/v RNS60 for 2 h in serum free media. Then immunoprecipitated chromatin fragments were amplified by semi-quantitative, (**b** CRE-distal; **c** CRE-proximal) and quantitative (**d** CRE-distal; **e** CRE-proximal) PCR as described under “Materials and Methods”. Results are the mean ± SD of three separate experiments. ^a^ *p* < 0.001 vs. control

Next, to understand whether CREB was directly involved in the transcription of myelin genes, we examined the recruitment of CREB to gene promoters by ChIP assay. Using Mat-Inspector V2.2 search machinery, we found two consensus CREs in the promoter of MBP gene (Fig. 5a). After immunoprecipitation of RNS60-treated oligodendroglial chromatin fragments using antibodies against CREB, we were also able to amplify 174 and 228 bp fragments from the MBP promoter corresponding to distal (Fig. 5b) and proximal (Fig. 5c) CREs (*p* < 0.001 vs. control), respectively. Because CREB-binding protein (CBP), a histone acetyl transferase, plays an important role in multiple CREB-mediated transcriptional activities, we investigated if CBP was also involved in RNS60-induced transcription of the MBP gene. As evident from PCR (Fig. 5b, c) and real-time PCR (Fig. 5d, e), RNS60 treatment induced the recruitment of CBP to the MBP gene promoter (*p* < 0.001 vs. control). In contrast, RNS60 was unable to recruit p300 to any of the CREs of the MBP promoter (Fig. 5b, e), suggesting that p300 was not involved in RNS60-mediated transcription of MBP in oligodendrocytes. Consistent to the recruitment of CREB and CBP to the CREs, RNS60 was able to recruit RNA polymerase (*p* < 0.001 vs. control) to the MBP gene promoter (Fig. 5b, e). These results are specific as we did not observe any amplification product either in control oligodendrocytes or NS-treated oligodendrocytes (Fig. 5b, e). Furthermore, no amplification product was observed in any of the immuno-precipitates obtained with control IgG (Fig. 5b, e).

**Fig. 6:**
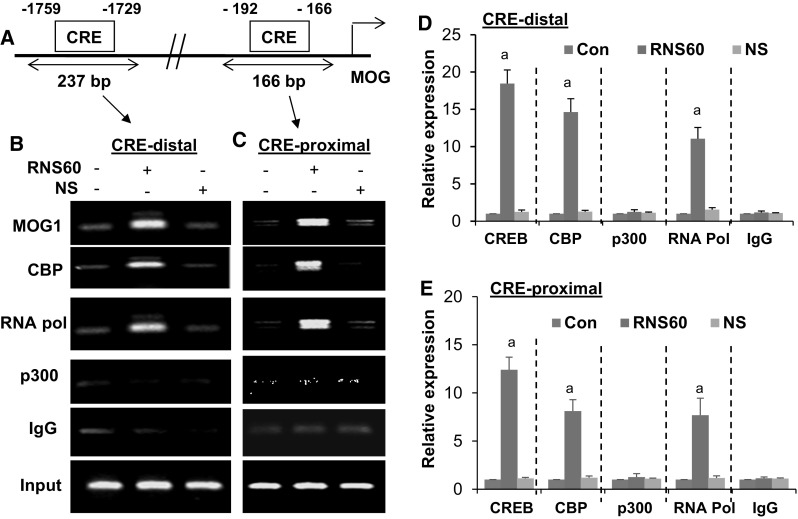
RNS60 treatment induces the recruitment of CREB to the MOG gene promoter in primary mouse oligodendrocytes. **a** Positions of two CREs in the mouse MOG gene promoter are shown. Cells were treated with 10% v/v RNS60 for 2 h in serum free media. Then immunoprecipitated chromatin fragments were amplified by semi-quantitative (**b** CRE-distal; **c** CRE-proximal) and quantitative (**d** CRE-distal; **e** CRE-proximal) PCR as described under “Materials and Methods”. Results are the mean ± SD of three separate experiments. ^a^ *p* < 0.001 vs. control

Similarly, consensus CREs are also present in the promoter of mouse MOG gene (Fig. 6a) and amplification of 237 and 166 bp fragments from the MOG promoter corresponding to distal (Fig. 6b) and proximal (Fig. 6c) CREs, respectively was found after immunoprecipitation of RNS60-treated oligodendroglial chromatin fragments with antibodies against CREB. Accordingly, RNS60, but not NS, also induced (*p* < 0.001 vs. control) the enrollment of CBP and RNA polymerase to both the CREs of the MOG gene promoter (Fig. 6b, e). Again, similar to that observed in MBP gene promoter, RNS60 remained unable to recruit p300 to the MOG gene promoter (Fig. 6b, e). Together, these results demonstrate that RNS60 alone is capable of increasing the recruitment of CREB and CBP to MBP and MOG gene promoters in oligodendrocytes.

### RNS60 Stimulates the Activation of CREB and Increases Myelin Gene Expression in Primary Mouse Oligodendrocytes via PI3K Pathway

**Fig. 7:**
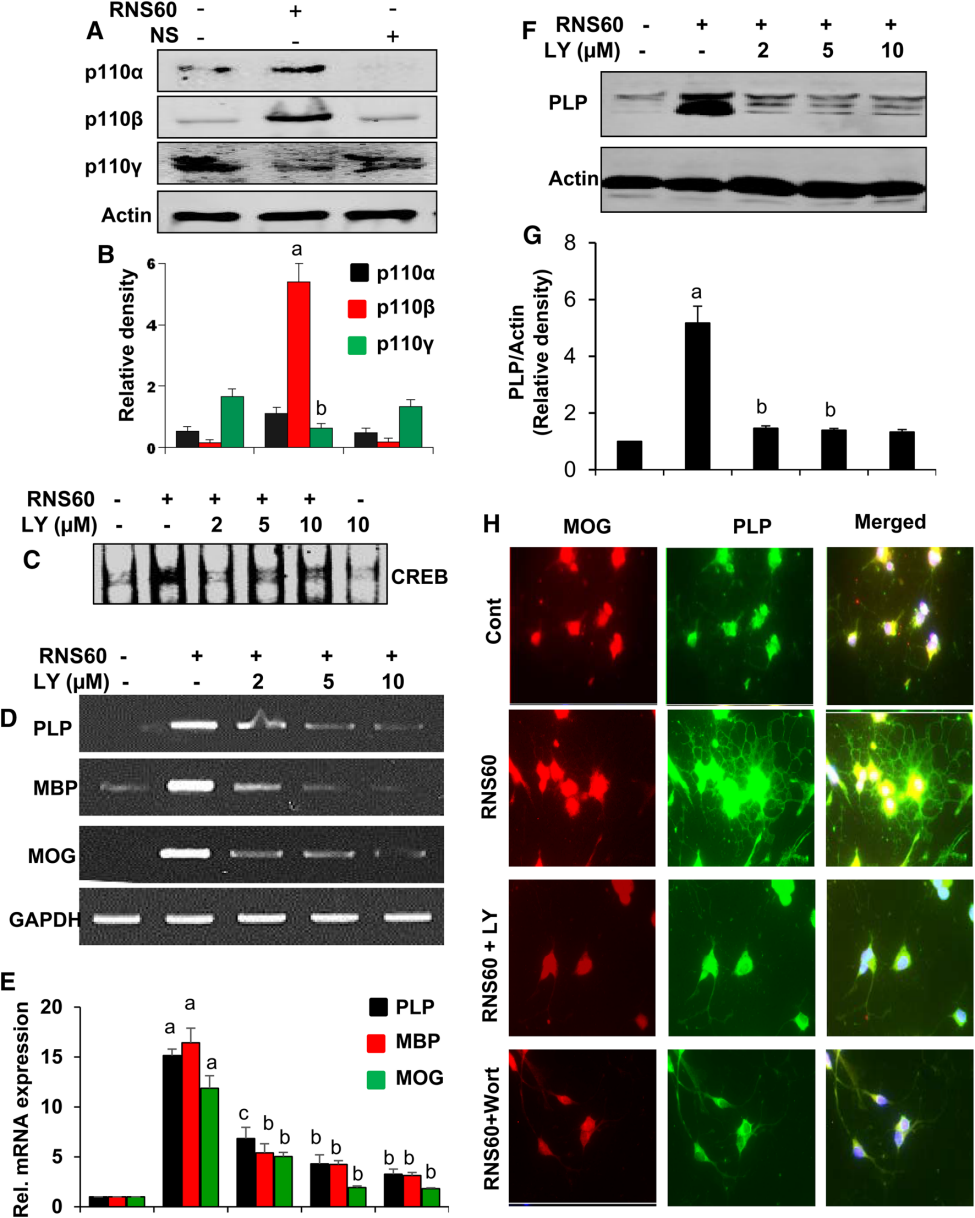
Role of PI3K in RNS60-mediated activation of CREB and expression of myelin-specific genes in primary mouse oligodendrocytes. Cells were treated with RNS60 for 30 min under serum free conditions followed by monitoring the levels of p110α, p110β and p110γ in membrane fractions by western blot (**a**). Bands were scanned and values are presented as relative density (**b**). Results are means ± SD of three different experiments. ^a^ *p* < 0.001 vs. control. Cells preincubated with different concentration of LY294002 (LY) for 30 min were treated with 10% v/v RNS60 under serum free condition. After 30 min of stimulation, activation of CREB was monitored by EMSA (**c**). After 6 h of stimulation, the mRNA expression of PLP, MBP and MOG was monitored by semi-quantitative RT-PCR (**d**) and real-time PCR (**e**). Results are means ± SD of three different experiments. ^a^ *p* < 0.001 vs. control; ^b^ *p* < 0.001 vs. control-RNS60; ^c^ *p* < 0.01 vs. control-RNS60. After 18 h of stimulation, the protein level of PLP was examined by western blot (**f**). Bands were scanned and values (PLP/Actin) are presented as relative to control (**g**). Results are means ± SD of three different experiments. ^a^ *p* < 0.001 vs. control; ^b^ *p* < 0.001 vs. control-RNS60; ^c^ *p* < 0.01 vs. control-RNS60. **h** Oligodendrocytes pre-incubated with either LY or wortmannin (wort) for 30 min were treated with 10% v/v RNS60 under serum free condition. After 18 h of treatment, cells were double-labeled with antibodies against MOG and PLP (**g**). Figures are representative of three independent experiments

Next, we examined mechanisms by which RNS60 increased the activation of CREB and upregulated myelin gene expression in oligodendrocytes. Recently we have demonstrated that RNS60 activates PI3K in microglial and neuronal cells [13, 32]. Therefore we investigated whether RNS60 involved PI3K for upregulation of myelin genes in oligodendrocytes. While in resting condition, subunits of PI3K are located mainly in cytoplasm, upon activation, these are translocated to the plasma membrane. Therefore after 1 h treatment with RNS60, we isolated plasma membrane from primary oligodendrocytes followed by monitoring the activation of class IA and IB PI3K by the recruitment of p110α, p110β and p110γ to the plasma membrane. Western blotting results from Fig. 7a, b showed that RNS60 specifically increased the recruitment of p110α and p110β to the membrane. On the other hand, RNS60 did not induce membrane localization of p110γ (Fig. 7a, b). In fact, RNS60 inhibited membrane localization of p110γ in oligodendrocytes (Fig. 7a, b). These results demonstrate that RNS60 is capable of specifically activating type IA PI3K p110α and p110β, but not type 1B PI3K p110γ, in primary oligodendrocytes.

Next, we examined the effect of LY294002 (LY), an inhibitor of PI3K, on RNS60-mediated activation of CREB and upregulation of myelin genes in oligodendrocytes. Inhibition of RNS60-mediated activation of CREB by LY (Fig. 7c) suggests the involvement of PI3K in RNS60-induced activation of CREB. LY294002 also markedly (*p* < 0.001 vs. RNS60) inhibited the RNS60-induced mRNA expression of PLP, MBP and MOG in primary oligodendrocytes (Fig. 7d, e). Western blot analysis of PLP (Fig. 7f, g) also corroborates this finding. Immunofluorescence analysis also indicated that wortmannin and LY294002 were able to suppress the expression of MOG and PLP in primary oligodendrocytes (Fig. 7h).

### Discussion

In MS, the intrinsic failure to repair and maintain myelin is believed to lead to axonal damage, causing disease symptoms and increasing disability. It is expected that by remyelination, nerves would be able to send proper signals again, restoring any loss of function as well as halting further damage. Therefore, delineating mechanisms to encourage remyelination is an important area of MS research [2, 12]. In the CNS, myelin is synthesized by specialized cells called oligodendrocytes and an increase in myelin-specific genes in oligodendrocytes is essential for remyelination [3, 5, 12, 29]. The current study highlights the stimulation of myelin-specific gene expression in oligodendrocytes by RNS60 that contains charge-stabilized nanostructures. RNS60 also stimulated the expression of myelin-specific genes in mixed glial cells without altering the expression of GFAP, an astrocyte-specific marker, suggesting that the effect of RNS60 is oligodendroglia-specific. This is unique because RNS60, a saline-based agent, is devoid of any active pharmaceutical ingredient, and hence is not targeted to a specific protein. Moreover, two important controls RNS10.3 (saline processed without elevated level of oxygen) and PNS60 (unprocessed saline with the same elevated oxygen concentration as RNS60) did not exhibit myelin gene stimulating effect, suggesting that the bioactivity of RNS60 is based neither on processing nor oxygen content alone, but a combination of the two.

Signaling mechanisms leading to the expression of myelin-specific genes are becoming clear. It has been reported that the master regulator cAMP response element-binding (CREB) plays an important role in myelination [29, 33]. Several lines of evidence presented here demonstrate that RNS60 upregulates the expression of myelin genes in oligodendrocytes via CREB. *First*, promoter of myelin-specific genes like MBP and MOG harbors several CREs. *Second*, RNS60 treatment alone induced the activation of CREB in oligodendrocytes. *Third*, siRNA knockdown of CREB abrogated RNS60-mediated upregulation of myelin genes in oligodendrocytes.

How RNS60 increases the activation of CREB in oligodendrocytes is an interesting question. Because of the facts that RNS60 may interact at the cell membrane, that activation of PI3K occurs in close association with cell membrane, and that RNS60 induces the activation of PI3K in microglia [13] and neurons [17], we were prompted to investigate the role of PI3K in RNS60-mediated activation of CREB and up-regulation of myelin genes. Here, we demonstrate that similar to neurons [17] and microglia [13], RNS60 induces the activation of p85α-associated p110α and p110β class IA, but not p101-associated p110γ class IB, PI3K in oligodendrocytes as well. Accordingly, pharmacological inhibition of PI3K suppressed RNS60-mediated activation of CREB and upregulation of myelin genes in oligodendrocytes. Earlier studies have shown that PI3K activation couples the CREB signaling pathway via Akt [34, 35]. Therefore, it is possible that RNS60-induced PI3K may couple CREB activation in oligodendrocytes via Akt.

In summary, we have demonstrated that RNS60 upregulates the expression of myelin genes in oligodendrocytes via activation of PI3K-CREB signaling pathway. Earlier we have shown that RNS60 treatment exhibited immunomodulation and ameliorated adoptive transfer of experimental allergic encephalomyelitis (EAE), an animal model of MS, via regulatory T cells [14]. Recently we have demonstrated that nebulization of RNS60 is capable of suppressing the disease process of EAE in mice [15]. One recent study has also shown that RNS60 increases spare glycolytic capacity of oligodendrocytes under normal culture conditions and enhances spare respiratory capacity of oligodendrocytes in the presence of a metabolic stress [36]. In the US, RNS60 has been approved by FDA for phase II clinical trials in MS and ALS patients. Although the in vitro situation of mouse oligodendrocytes and oligodendrocytes in culture and its treatment with RNS60 may not truly resemble the in vivo situation of these cells in the brain of MS patients, our results highlight important myelinogenic property of RNS60 and indicate that RNS60 may have therapeutic promise in MS and other demyelinating disorders.
